# Agricultural Science in the Wild: A Social Network Analysis of Farmer Knowledge Exchange

**DOI:** 10.1371/journal.pone.0105203

**Published:** 2014-08-14

**Authors:** Brennon A. Wood, Hugh T. Blair, David I. Gray, Peter D. Kemp, Paul R. Kenyon, Steve T. Morris, Alison M. Sewell

**Affiliations:** 1 Institute of Agriculture and Environment, Massey University, Palmerston North, New Zealand; 2 Institute of Veterinary, Animal and Biological Sciences, Massey University, Palmerston North, New Zealand; 3 Institute of Education, Massey University, Palmerston North, New Zealand; Northwestern University, United States of America

## Abstract

Responding to demands for transformed farming practices requires new forms of knowledge. Given their scale and complexity, agricultural problems can no longer be solved by linear transfers in which technology developed by specialists passes to farmers by way of extension intermediaries. Recent research on alternative approaches has focused on the innovation systems formed by interactions between heterogeneous actors. Rather than linear transfer, systems theory highlights network facilitation as a specialized function. This paper contributes to our understanding of such facilitation by investigating the networks in which farmers discuss science. We report findings based on the study of a pastoral farming experiment collaboratively undertaken by a group of 17 farmers and five scientists. Analysis of prior contact and alter sharing between the group’s members indicates strongly tied and decentralized networks. Farmer knowledge exchanges about the experiment have been investigated using a mix of quantitative and qualitative methods. Network surveys identified who the farmers contacted for knowledge before the study began and who they had talked to about the experiment by 18 months later. Open-ended interviews collected farmer statements about their most valuable contacts and these statements have been thematically analysed. The network analysis shows that farmers talked about the experiment with 192 people, most of whom were fellow farmers. Farmers with densely tied and occupationally homogeneous contacts grew their networks more than did farmers with contacts that are loosely tied and diverse. Thematic analysis reveals three general principles: farmers value knowledge delivered by persons rather than roles, privilege farming experience, and develop knowledge with empiricist rather than rationalist techniques. Taken together, these findings suggest that farmers deliberate about science in intensive and durable networks that have significant implications for theorizing agricultural innovation. The paper thus concludes by considering the findings’ significance for current efforts to rethink agricultural extension.

## Introduction

The demand for agricultural transformation is both urgent and worldwide [1], [2]. Among others, farmers are now routinely called upon to begin practising new forms of knowledge. The prevailing approach to agricultural learning identifies this new knowledge as something that farmers do not possess and that therefore must somehow be transferred to them. Knowledge is typically equated with objective content and its movement accordingly reduced to the one-way transfer of technology. In the linear model, research problems are set and resolved by scientists who develop technologies in laboratories and research stations; these technologies are then passed on to extension agents for transfer to farmers. Whatever its successes may have been in the past, it is generally agreed that the technology transfer model is poorly equipped to deal with the complexity and riskiness of the problems now faced by farmers [3].

Some 20 years ago a powerful alternative to technology transfer emerged that radically prioritized farmer participation in the development of knowledge [4]. Over the intervening years however, these Farmer First initiatives have achieved limited successes and more recently the search for alternatives has turned to the theory of agricultural innovation systems [5], [6]. On these terms, innovation is theorized as the complex outcome of open-ended interactions between agriculture’s various stakeholders, including for example farmers, merchants, extension agents, scientists and government officials [7], [8]. Such theorizing about agricultural innovation emphasizes the role played by open-ended networks with heterogeneous members. In many respects then, the theory of agricultural innovation systems is a recent variant of the long-running diffusion of innovations paradigm, which originated in post-war rural sociology but has since evolved into an interdisciplinary field that similarly emphasizes the importance of network principles [9], [10], [11].

Whereas Farmer First initiatives had radically prioritized farmer participation, recent theorizing about agricultural innovation systems emphasizes the critical role played by heterogeneous relationships. Recent research focusing on farmer networks of practice returns a similar result; the business of farming embeds farmers in influential relationships with an occupationally diverse array of people [12], [13]. These new emphases on actor diversity and system complexity have often been accompanied by calls to professionalize network facilitation [6], [12], [13]. As the task of facilitation is seen as too demanding for any of the actors in their ordinary capacity, it is believed to be more effective when undertaken by specialists. Such claims risk reproducing the very gap between farmers and new knowledge that animates the technology transfer model. With its well-established focus on credentialed skill and social closure, the professional project may diminish both the contribution of farmers and the openness to alternatives that innovation requires [14].

Innovation systems theory argues for the importance of making knowledge accessible, both in terms of production and dissemination [15]. Innovation results from interactions between diverse, self-organizing actors and the complexity of their interaction makes the knowledge they produce highly unpredictable. Rather than attempt to control such open-ended interactions, systems researchers have called for a reconceptualization of how these networks are facilitated [8]. As they have noted, the further development of innovation theory requires empirical investigations that address three critical gaps in our knowledge [16], [17], [18]. We need further research on the facilitation of agricultural networks as a specialized function. As innovation is an outcome of open-ended interactions between such diverse actors as scientists and farmers, we need to know more about how agriculture’s local research stakeholders are organized. Finally, the role of communication must be systematically reconceptualized and to date we lack sufficient research on the cultural practices that inform agricultural networking. This paper addresses these three gaps in our knowledge.

This paper contributes to innovation systems theory by exploring the significance of the networks in which farmers discuss science. Conceptually, the existence of these networks implies that individual farmers and scientists are interrelated; they figure somehow in each other’s ego networks. To put the point more empirically, farmers and scientists meet and on occasion work together. On such occasions, farmers participate as local research stakeholders in agricultural science. In this paper we explore how such farmer participation embeds science in the complex networks of agricultural innovation systems. We have undertaken a three-year research project designed to examine knowledge-sharing relationships between science and farming. The project centres on a pastoral farming experiment collaboratively undertaken by a group of five scientists and 17 farmers. Here we report our findings with regard to how the 17 farmers networked this experimental science with the larger field of contacts they make to share and enhance their agricultural knowledge.

By examining the embedding of science in farmer networks of practice, we aim to help re-conceptualize the roles of facilitation and communication in agricultural innovation. Our analysis of these farmer networks of practice differs markedly from previous research. Farmer networks are typically conceived as a coordination of abstract functions rather than as interactions between social individuals [12], [13]. The networks produced by such analyses are diffuse and weakly organized, a result that has prompted calls for more professional facilitation. However, such abstract networks belong to no-one in particular and they should not be mistaken for farmer constructions. Rather than focus on generic or functional roles, we propose to investigate farmer networking as an interpersonal practice. When farmers directly contact other individuals, how do they exchange agricultural knowledge? Our findings suggest that farmers exchange knowledge in densely tied and strongly organized interpersonal networks. These networks decisively shape the communication of agricultural science in ways that limit professional closure and effectively disable the linear transfer of technology. We therefore conclude by considering the findings’ general significance for current efforts to rethink how agricultural innovations are produced and disseminated.

## Method

### Research Objectives and Participant Selection

This study has three objectives. We seek to find out how farmers participate with agricultural scientists as local research stakeholders and how they extend this participation by sharing knowledge with other people, focusing specifically on the role of facilitation and communication in these networks. To meet these objectives, our study requires farmer participants who are local research stakeholders. How are they to be selected? Probabilistic sampling has decided advantages. By controlling for extraneous factors, random selection allows for robust generalizations to populations of interest. Network analysis undertaken on these terms is called ego-centric and it is important to note its methodological limits [19]. An interpersonally related population of farmers and scientists has collective structures that connect its members together. Clearly, network facilitation and communication occur not only within but also across each individual’s ego-centric domain. Such group-level processes cannot be analysed on the basis of randomly selected individuals because such selection randomizes the very relationships that tie these egos together [20]. Meeting our objectives thus requires group-level data and sociometric analysis [19]. Rather than draw a random sample, sociometric analysis must go more directly to a bounded population of theoretical interest and collect data from all its members [21]. This is the strategy adopted here.

We have assembled a group of five agricultural scientists and 17 farmers using the following procedures. Having resolved to study knowledge sharing between scientists and farmers, the co-authors established a three-year experiment on a 7.5 hectare university farmlet. As outlined further below, the experiment was designed to produce new knowledge about herb-based pastures in collaboration with farmers. A number of purposes informed the selection of these farmer participants. Farmers were selected to ensure a mix of herb pasture experience, ages and farm systems, including significant climatic variation. This ensured that the farmers brought a wide range of stakeholder knowledge to bear upon the experiment. More significantly for the purposes of this paper, the farmers were known personally by the five agricultural scientists on the project team (HB, DG, PDK, PRK, SM). Over numerous years, these scientists have worked closely with many local farmers and the study participants were drawn from these prior contacts. This selection process is clearly non-random and so does not permit statistical generalization. However, our objectives require sociometric data and this necessitates network-based selection rather than either randomized or convenience sampling. Accordingly, we have identified 17 farmers who are interpersonally connected with five scientists and drawn this grouping together to investigate its cohesiveness and to assess the theoretical significance of networking by its farmer members.

### Research Setting

The study centres on a 2011–2014 farmlet experiment that investigated lamb finishing on herb and legume-based pastures composed of red and white clovers (*Trifolium pratense* and *T. repens*), chicory (*Cichorium intybus*) and plantain (*Plantago lanceolata*). This experiment was designed to produce new scientific knowledge. Although previous research on ewes and lambs had identified significant finishing gains over one season, the performance of herb pastures in a year-round farm system had not been scientifically established [22]. The experiment is of interest not only to agricultural scientists but also to New Zealand farmers, many of whom have been considering or trialing these new pastures. Moreover, although the farmlet is considerably smaller than a commercial operation, it is of sufficient size to operate as a scalable system. Running the experiment for three years thus involved many familiar farming activities, such as lamb purchase and sale, the management of stocking density and rotation, and the use of drenches, fertilizers and herbicides. The farmlet is also subject to the same weather vagaries as commercial farms nearby. The 17 farmer participants visited the herb experiment four times a year, reflecting the various seasons and physiological states of animals and plants. Experimental results to date were regularly considered and grazing decisions reviewed and determined through collaborative discussion by the group as a whole. These regular meetings at the farmlet were also accompanied by a range of other activities. Typically, visits to the experiment included over-night stays for the farmers, leaving ample room for informal socializing. The farmers participated in a series of science-oriented workshops and talks on topics relevant to pastoral farming (such as animal autopsies, food science, freshwater ecology, parasite control, precision agriculture and weed management). The group also visited the farms of members who have their own trials with herb pastures underway.

The setting described above is designed to position farmers as local and personal stakeholders in the development of agricultural science. The collaborative management of the experiment and the various attendant activities produced numerous knowledge-sharing interactions between the group’s farmer and scientist members. Data on these small-group interactions have been collected using of mix of field observation and interview techniques. We have reported the results of this investigation elsewhere [23]. In brief, our findings show that the 17 farmers acted as local stakeholders by forming a community of practice with the five scientists. This community is constructed by small-group processes that align scientific concepts with farming experience and connect scientific experimentation with the unique situations of individual farmers. In this paper we extend these findings by examining how the farmer-scientist community has been elaborated in farmer networks of practice. As will be seen, the results of this investigation reveal similar processes that connect farmlets to farms by aligning concepts with experience. Here we focus specifically on the networking dimensions of such processes. It should be noted that the research setting is likely to have affected the network data collected. We may hypothesize for example that the small-group integration of farmers as stakeholders has increased their ability to communicate about the experiment in their personal networks. Our design lacks the controls required to test such hypotheses statistically. However, as noted above our objective is conceptual development rather than empirical generalization. Exploring the significance of farmer networked science for agricultural innovation theory requires not only the selection but also the mobilization of farmers as local stakeholders and this is what the research setting achieves.

### Data Collection and Analysis

In order to investigate the networks in which farmers discuss science, we have adopted a mixed methods design [24]. Personal interview surveys of all 22 group members have been used to collect sociometric data for the quantitative analysis of knowledge exchange networks; free-form interviews of the 17 farmer participants have been used to collect data for qualitative analysis of the perspectives on knowledge that inform farmer networking.

A range of sociometric data has been collected from the 22 farmers and scientists who make up the group under investigation. Using a mix of name-generator and roster formats, four surveys have been undertaken [19]. Prior to the first on-site visit to the experiment in mid-2011, each farmer was interviewed at home to identify their pre-existing contacts for herb pasture knowledge. Immediately following the project’s launch, the 17 farmers and five scientists were individually surveyed to identify who of the other group members they knew personally before the project began. In December 2012 follow-up interviews were undertaken with the farmers to identify who they had talked to about the pastoral farming experiment over the preceding 18-month period. Finally, in early 2013 the 22 group members were presented with a roster of all the individuals named in previous farmer surveys and asked to identify all those they knew personally. These survey data have been analysed using Ucinet software [25]. The various network metrics are described in context along with the results they produced in the first half of this paper.

Prior to the first on-site meeting in mid-2011, the 17 farmers were also interviewed to gather qualitative data on their perspectives about knowledge exchange. These interviews were undertaken at the farmers’ homes by the project team’s sociologist (BW) and ranged from 45 minutes to two hours in duration. The interviews used an open-ended format and focused on the qualities of the people whom the farmers rated as their most valuable sources of knowledge. The farmers were asked to select from a list of their existing contacts the three they had found ‘the most useful to talk to in order to get hold of or share information and ideas’ about herb pastures. They were then invited to discuss why they had singled out these contacts and the transcripts of these conversations were coded by the sociologist using NVivo 9 software [26].

Initial coding was undertaken to isolate a field of statements that recorded judgements about the value of knowledge exchange. The great bulk of these statements relate personal experiences, although on occasion the farmers also offered more general opinions. These statements were further coded using the iterative procedures of constant comparison [27]. This second phase grouped the statements into higher-order themes representing the values farmers commonly ascribe to herb pasture knowledge [28]. The thematic analysis identified three broad value clusters: (1) knowledge delivered in person, (2) the sharing of farmer experience, and (3) farmer empiricism. The initial thematic analysis was reviewed and revised by the project team as whole, which includes agricultural scientists who have considerable experience working with farmers. The validity of the three themes has been further confirmed by member checking with the 17 farmers, including feedback on initial findings presented during on-site meetings. The thematic analysis results are presented in the second half of this paper.

### Ethics Statement

The study conforms with the principles of the 1964 Declaration of Helsinki and was conducted in accordance with Massey University’s Code of Ethical Conduct for Research, Teaching and Evaluations Involving Human Participants (http://www.massey.ac.nz/massey/fms/Human%20Ethics/Documents/MUHEC%20Code%202013.pdf). As required by University procedures (http://www.massey.ac.nz/massey/research/research-ethics/human-ethics/forms-and-procedures.cfm), the study was independently peer reviewed and a Screening Questionnaire was completed. This process determined that the study was low risk for all participants and thus did not require further approval by the Massey University Human Ethics Committee (MUHEC). A signed Screening Questionnaire has been lodged with MUHEC and the study (‘Farmer Learning Project’) is registered on the Committee’s 2011 Low Risk Database. All participants signed informed consent forms prior to taking part in the study. The consent documents informed participants of the study’s low risk status and gave confidentiality undertakings that no participant would be individually identifiable in any public presentations of the findings.

### Network Analysis Results

We begin by considering the network of prior acquaintances that existed between the farmers and scientists before the launch of the experiment in 2011. All the 17 farmers were known by at least one of the five scientists. Group-level analysis has been undertaken to measure how these prior acquaintances positioned the farmers as research stakeholders in terms of group cohesiveness and network power. Following this analysis we investigate the development of the 17 farmers’ contacts for herb pasture knowledge over the first 18 months of the experiment. This egocentric analysis is then extended by investigating the sharing of farmer contacts at group-level. Taken together, these analyses reveal the character of farmer networking about the experiment, including the sorts of people it reaches and how such reach is facilitated.

### The Prior Acquaintance Network

**Figure 1:**
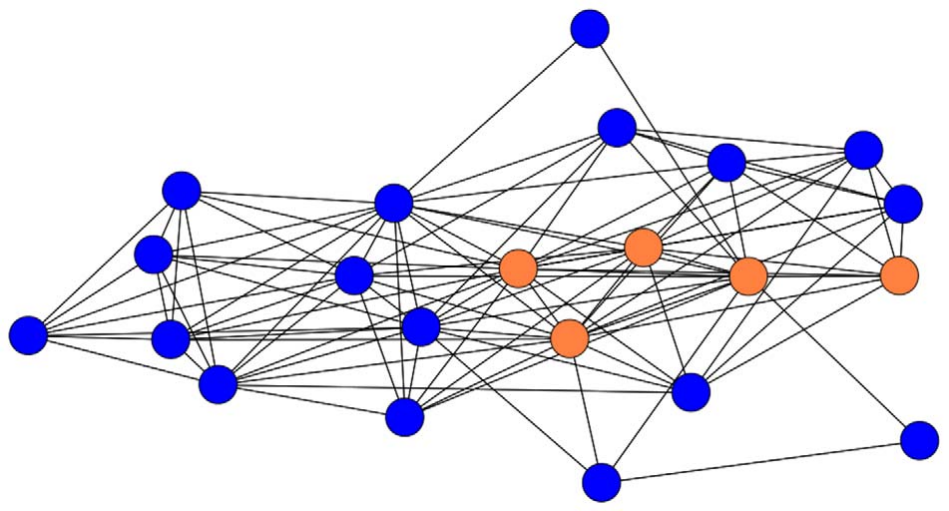
The network of prior contact between the group’s 22 members. Red nodes are the five scientists, blue are the 17 farmers. The figure was produced using Ucinet’s Netdraw application.

The network of prior acquaintance between group members has been analysed to determine how it positions the farmer participants as stakeholders in the exchange of knowledge. Prior contact may be regarded as a communication resource that is distributed between the group’s 22 members and some may hold more of this resource than others. Hypothetically for example, the network may structurally privilege the five agricultural scientists over the 17 farmers; the former may personally know many while the latter know relatively few. This hierarchical situation would result in a network centred on highly connected scientists radiating ties out to the more dispersed farmers. Such a structure might perhaps be expected from the scientist-driven recruitment process described above. However, the network of prior acquaintance between the group’s 22 members does not exhibit this sort of centralised structure (Figure 1).

As Figure 1 shows, the group’s prior contact network is not centred on a few scientists who have brought an assortment of farmers together, many of whom are unknown to each other. A dense thicket of ties binds the acquaintance network together, creating the impression of a flat rather than hierarchical structure. The structural metrics reported in Table 1 confirm this interpretation.

**Table 1:** The network of prior acquaintance between the group’s 22 participants.

network density	0.44
average shortest path	1.6
betweenness centralization	11.6%

Table 1 reports on three structural dimensions of the group’s prior acquaintance network. Density is the number of actual ties divided by total possible ties, with a score of 0 meaning that none of the actors are tied together and a score of 1 that everyone is directly connected [19]. As it measures the extent of actor ties, density is taken to indicate network cohesiveness. There were 102 prior ties between the group’s 22 members, giving a density of 0.44. As density scores vary by network type they cannot be interpreted as high or low in absolute terms. However, when the group members first gathered in 2011 they were like a first-day class in which on average everyone knows half their classmates; it was clearly not a roomful of strangers. This suggests a cohesive network with many personal connections. Such cohesiveness is also indicated by the length of the average shortest path. Actors who know each other directly are one degree apart; if they know each other only indirectly through another then they are two degrees apart and the more others it takes to connect them the further apart they are. In network terms, the shorter the path between two actors the more efficiently they can communicate [19]. The average shortest path between all pairings of the 22 members is a low 1.6, suggesting that everyone in the acquaintance network is close together and that group members can communicate with each other relatively easily.

Although the density and shortest path results indicate a cohesive network, this does not necessarily mean an absence of hierarchy. For example, a subset of members might hold the majority of ties and it is this subset’s connectivity that draws everyone together. Such actors have the advantages that come with being more centrally positioned. Network centrality can be measured in a number of different ways and here we focus on betweenness in particular [19]. Betweenness centrality refers to the number of times an actor falls on the shortest path linking two other actors together. By falling between otherwise unconnected pairs, an actor is positioned to control the flow of information. Betweenness centrality can be calculated at the level of the whole network as the average difference between the score of the most central actor and all the others. In percentage terms, the index varies between 100, in which one actor holds all the ties, and 0, in which everyone is directly connected and thus there is no centrality in the network. The prior acquaintance network’s betweenness centralization is 11.6%, suggesting relatively little difference between the 22 group members’ ability to control the flow of knowledge. The network is decentralized; its cohesiveness does not rely on a few highly pivotal players making connections between otherwise isolated actors.

### Farmer-Driven Networking

The 17 farmers were surveyed prior to the first meeting of the experiment’s group in order to identify their pre-existing herb knowledge contacts. Some 18 months later, they were re-interviewed to identify both the prior contacts and previously unmentioned people with whom they had discussed the herb pasture experiment. The farmers were also asked to identify the occupations of all the contacts they identified. These egocentric survey data have been analyzed to indicate how the 17 farmers networked knowledge about the experiment by activating and adding to their existing interpersonal ties. Table 2 summarises these processes by comparing the occupational profile of the farmers’ contacts before the experiment began with the contacts’ occupational profile 18 months later.

**Table 2:** Farmer contacts for herb pasture knowledge by project stage and occupation.

Contact occupation	Prior knowledge contactsbefore the project began	Prior contacts informed about theexperiment at 18 months	New contacts identifiedat 18 months	Project contact networkat 18 months
Accountant	0	NA	1	1
Banker	0	NA	5	5
Consultant	8	5	1	6
Contractors	12	4	3	7
Farmer	63	46	80	126
Industry good	4	2	5	7
Merchant (fertiliser)	5	2	1	3
Merchant (seed)	20	12	4	16
Other	2	1	4	5
Scientist	8	5	2	7
Veterinarian	3	2	7	9
Total	125	79	113	192

By 18 months after the experiment’s launch, the 17 participating farmers had discussed the farmlet with 63.2% (79) of their pre-existing contacts and with 113 new individuals not previously identified. These results suggest significant network growth. At 18 months the farmers identified 192 contacts, 53.6% more than when the experiment began. The results also clearly show that the farmers made contact with their social peers more than with anyone else. Half (63) of the 125 prior contacts were fellow farmers, three times the commitment to any other occupational group. At 18 months, 73.0% (46) of these prior farmer contacts had been talked to about the experiment. Moreover, 70.8% (80) of the new contacts identified were other farmers. Seed merchants are in distant second place, comprising 16.0% (20) of prior contacts and 8.3% (16) of the farmers’ ego networks at 18 months. They are followed by a mix of other occupations at 0–10% and 1–5% respectively.

**Figure 2:**
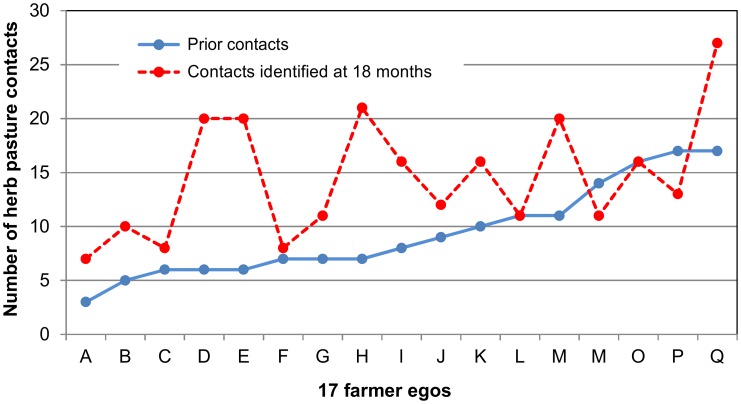
Degree distributions of the 17 farmers’ prior contacts and the contacts made after 18 months. Data is plotted by ascending size of the farmers’ prior contact networks.

Fellow farmers were networked much more than any other group. This result has been analysed in more detail at the level of the farmers’ individual contact networks. Each of the 17 farmers has their own personal cluster of contacts and these ego networks vary widely by size. Figure 2 plots the degree distributions of both the prior contacts and the contacts that had been made about the herb pasture experiment after 18 months. Before the experiment began, on average each farmer had 9.4 contacts; 18 months later they had 14.5. This increase in network size is not a uniform process. The number of prior contacts ranges from 3 to 17, the number of contacts at 18 months from 7 to 27. Growth rates have been calculated for the farmers’ ego networks over the 18 month period. Analysis shows that these rates are significantly correlated with the number of prior knowledge contacts (r^2^ = 0.370, p = 0.009, slope = −11.58). As Figure 2 suggests, the smaller networks tended to grow more than the larger networks.

Further analysis suggests that social structure also plays a significant role in the development of the farmers’ networks over time. The density and occupational heterogeneity of the 17 ego networks have been calculated. As above, density is actual divided by possible ties and so requires data for all actors in each network. As is conventional, this data was collected by asking the farmers to report who of their contacts knew each other personally [19]. Occupational heterogeneity has been calculated using Blau’s heterogeneity index, which is measured as 1 minus the sum of the squares of the proportions of each occupational category in the ego networks [29]. The index varies between 0, where all contacts have the same occupation, and a maximum that approaches 1, where contacts are evenly spread across the occupational categories (the maximum value is determined by the number of categories (k) and equals (k−1)/k; thus with 11 occupations the maximum is 0.91). While density indicates network cohesiveness, the heterogeneity index measures the diversity of information sources used by each farmer. The density and heterogeneity scores have been correlated with the growth rates of each farmer’s network over the 18-month period. The linear regression of network growth on density is significant (r^2^ = 0.357, p = 0.011, slope = +284.00) and so too is the regression of growth on heterogeneity (r^2^ = 0.342, p = 0.014, slope = −459.87). Analysis thus suggests that the farmers’ networking was driven by sociological traits. The denser and more occupationally homogenous a network, the more it grew.

### The Sharing of Alters

The networks of the group’s 22 members exist in isolation only in abstraction; in reality they intersect. The 17 farmers share contacts directly when they identify the same individual in their ego networks. Moreover, one of the farmers may personally know individuals in the other farmers’ networks even though they have not identified them among their own contacts. The five agricultural scientists may also personally know individuals nominated by the farmers. To use network terminology, the group’s 22 ego networks intersect because they share alters. The surveys identified 238 alters in total and 85.3% (203) of these are known personally by more than one ego. When a contact is known by two of the participants, a triadic structure is formed. Triads tie three actors together in the smallest structures of a group life that operates beyond the level of the individual [30]. Triadic structures produce collective power in a number of different ways and three structures are of particular interest here.

The farmer participants’ 238 alters can be shared (1) by two of the participating farmers, (2) by two of the scientists, or (3) by a farmer and a scientist. These three triadic forms have been identified using Ucinet’s brokerage routine [25]. As threesomes, these triads create opportunities for the 238 contacts to learn about the herb experiment from multiple group members. Table 3 analyses the distribution of these opportunities between the group’s 17 farmers and five scientists. If the network privileges multiple messaging by scientists, then alters will be most frequently embedded in triads with two of the scientists. The ability of the farmers to deliver multi-member messaging is indicated by the relative significance of the other two triadic forms. Table 3 cross-tabulates the three triads by alter occupation. This analysis indicates the extent to which the 22 group members’ ability to collectively reinforce knowledge varies according to the occupation of the person addressed.

**Table 3:** Group contact sharing by triadic form and alter occupation.

Alter occupation	Shared by 2 farmers	Shared by 2 scientists	Shared by mixed pair	Total
Accountant	1	0	0	1
Banker	15	1	4	20
Consultant	253	42	237	532
Contractor	77	16	53	146
Farmer	1263	116	757	2136
Industry good	102	25	115	242
Merchant (fertiliser)	52	0	9	61
Merchant (seed)	240	2	52	294
Other	36	0	0	36
Scientist	96	32	128	256
Veterinarian	199	23	163	385
Total	2334	257	1518	4109

As Table 3 shows, the sharing of alters produced 4109 pairings of the group’s 22 members (on average 20 ego pairs per alter). Nearly ten times as much alter sharing takes place between the 17 farmers (2334) as between the five scientists (257). The scientists share herb contacts six times more frequently with the farmers (1518) than they do with each other. The significance of farmers is also evident in the distribution of alter occupations. Farmer alters make up half (2136) of the total shared contacts, four times more frequent than the second-placed private consultants (532). Across all three triadic forms, these farmer contacts are shared three to five times more frequently than any other occupational role. The results also reveal some minor differences between the scientist and farmer members. Relative to the farmer-only triads, the scientists are less likely to share merchant contacts and more likely to share consultants, industry good representatives and fellow scientists. In general however, the sharing of farmer alters by the 17 farmer egos is clearly the group’s dominant triadic form.

The extent of alter sharing indicates that the group’s networks generate numerous communication opportunities. For example, 143 farmer alters have been identified in total (see Table 2). These farmer alters are embedded in 2137 triads with the 22 members (on average each is located between 15 ego pairings). Collectively then, the group is strongly positioned to reinforce knowledge of the herb pasture experiment with messages from more than one group member. Triadic analysis shows that the 17 farmers are positioned to deliver these multi-member messages more frequently than the five agricultural scientists. Moreover, reflecting their numerical dominance in the alter population, farmers are much more likely to receive such messages than any other occupational grouping.

### Thematic Analysis Results

The above analyses have shown how interpersonal contacts tie the group of farmers and scientists closely together and consolidate ties with others further afield. In this section of the paper we turn to a qualitative analysis of the communication practices that inform farmer networked science. We analyse what knowing someone means for the group’s farmer members and how these meanings shape the way they exchange knowledge. We focus in particular on farmer practices because, as the above results show, farmers are the most prominently positioned actors in the group’s networks. As outlined in the Methods section, the 17 farmer participants have been interviewed about their most valuable contacts and the resulting statements have been thematically analysed to reveal the values ascribed to knowledge exchange. Three general themes have been identified: (1) the value of knowledge delivered in person, (2) the sharing of farmer experience, and (3) farmer empiricism. Along with minor differences and variations, all the farmers strongly endorsed these values. Here we present the results of this analysis, with thematic generalization accompanied by exemplary farmer statements.

### Knowledge Delivered In Person

The farmers’ statements below show that they regularly contact numerous other people to secure the farming resources they need. This practical networking sustains wide-ranging and durable relationships that emphasize the interpersonal value of knowledge exchange.

*When you’re managing a farm you can’t be an expert on everything. That’s not the way it is. You rely on a host of people to advise you.* (Farmer A)

*I don’t like the idea of jumping from one guy to the other and just chasing the best deal all the time. You try and form a relationship with them so that you can trust them. And generally, as I say, he wouldn’t still be here after how many years, 12 years, it’s longer than that now, it’s 13, I wouldn’t still be using him.* (Farmer B)

*Oh he comes past here every Monday and sometimes calls in. It depends who’s got the scones and the pikelets ready. You know, he does this area on Mondays. But he just comes and he’ll sit and talk about his soccer team more than anything because he runs the local soccer team and he’s fanatical on soccer.* (Farmer C)

*I can ring and have a chat and ask him questions about whatever anywhere anytime sort of thing, just because we’re mates. That’s what friends are for.* (Farmer D)

*Because he’s an enthusiast about his subject and he will take me into places where others won’t. Because he doesn’t act as an advisor, he’ll just, exactly like me at the moment, he’ll rave on at great length about his subject.* (Farmer E)

As the farmers routinely seek knowledge from others, such calls often result in long-term relationships. Some of the older farmers had worked with the same seed merchant and veterinarian for over 20 years. Long-term relationships inevitably become highly personal. Rather than being narrowly defined by any formal role, they thicken with social interaction and sentiment. Contacts are thus expected to speak well beyond the bounds of any specialized competency. The value that farmers place on richly interpersonal contact means that their most valued relationships are multi-dimensional and their knowledge networks are highly informal.

### Sharing Farmer Experience

The 17 farmers all emphasized the value of knowledge sourced in farming experiences that are both actual and ongoing. As the following statements show, they primarily make contact with fellow farmers and other agricultural individuals in order to share such experience-based knowledge.

*I suppose every time you go to a function or anything else you’ve got to have something to talk about. You know farmers, they generally talk about the farm and not much outside the farm gate.* (Farmer F)

*Every time I see the seed rep he talks to me of new products, and the first thing I ask him is ‘who’s growing it?’ Because I’m more interested in who’s actually growing it so I can actually find out why they’re growing it and hear what their experiences are rather than what the seed rep’s actually telling me. He’s my contact man as to who’s doing it.* (Farmer G)

*A group of us went down to [a lucerne farm in the South Island]. We did it specifically to get some ideas for us. For us using that lucerne system’s not feasible but what he’s done has applicability. The suggestion came from my neighbour and a group of about seven of us [farmers] went down just to see what he was up to. Danny rang us all up and said, ‘hey are you interested in coming with us down to Doug’s for lucerne?’* (Farmer A)

*They kept [the discussion group] round about 15 farmers roughly. So you didn’t go round the farm too often, like it’s one a half years when you’re back on the farm. Some of them were a waste of time and some of them were quite good.* (Farmer C)

When farmers make contact with others, they like to talk about farming. Such talk is not abstract; it shares experiences about individual farms. Indeed, all herb pasture contacts, farmers and non-farmers alike, are valued for their ability to relay experiences from other farms. The farmers also exchange knowledge directly by visiting each other’s properties. In making these visits, they seek learning they can personally transport back to their own farm. In general then, both in their discussion groups and more impromptu gatherings, the 17 farmers primarily value knowledge exchanges that are based on the sharing of farmer experience.

### Farmer Empiricism

In keeping with their emphasis on the value of farming experience, the farmer statements esteem knowledge of the particular over knowledge in general. Philosophically speaking, this emphasis on the particular favours knowledge exchanges based on empiricist principles.

*Because using a situation where there’ll be chicory mix or anything really, it is typical to your property and unique to you and your style, so, you know, an internet can’t really cater for that.* (Farmer H)

*I think basically you see what’s going on over the fence and if you’re interested you’ll ask, you’ll see what works and what doesn’t. Basically in the same land, if it works there it works for us.* (Farmer I)

*Some of the innovative younger ones like the boy Smith, he was trying it, wasn’t he? And that’s coastal area compared to here. I mean there’s quite a difference in management and everything else.* (Farmer J)

*15 years ago we tried chicory by itself on a four ha block and it was not a success. We’d heard about the product but we had no management skills and there was no one around to glean information from. We were silly – it was a bad site and just too small. Then for a period of ten years every year we put one kilo of chicory in our grass mix. The chicory came through but being summer dry we had a thistle problem and they’re not tolerant of thistle sprays. But even now we’ll close the paddock up for a while and the chicory will come away. But grazing management, yeah, just doesn’t work in this type of farming, in grass, because you tend to graze too low. We’re learning. Chicory does need that on-off grazing. We’re still trying to refine that because we’re still maybe leaving it a bit too long. And we’re learning things like you don’t graze in the rain and things like that, when it’s wet. A big learning curve.* (Farmer C)

*Just farmers talking about using chicory and how great it was for growing lambs. But it’s always been around and then suddenly it’s like, hang on, why aren’t we going to give it a go?* (Farmer K)

As the above statements illustrate, the farmers seek knowledge that can be applied to their individual farm by contacting individuals who can share the experiences of other equally individual farms. This overriding concern to know the particular puts a strong brake on generalization. One farmer described his knowledge as *tiny things picked up from everywhere* (Farmer I). This sort of knowledge is produced by empiricist rather than rationalist procedures. Whereas rationalists interpret cases by applying general rules, for empiricists such rules have limited applicability because no two situations are ever identical. Rather than uniformly falling beneath some covering law, situations always differ in some discernible way and in the future they are likely to become more or less different in ways as yet unforeseen [31]. Such empiricist commitments favour distinctive learning skills, notably reasoning by way of analogy [32]. The farmers move experience across farms by comparing and contrasting examples. The drawing of these analogies produces valid knowledge by persistently and skilfully finding informative similarities and differences. Producing such knowledge requires an ability to recall and recount experiences over considerable periods of time. By drawing analogies and recounting experiences, the farmers are thus able to exchange what collectively amounts to their already substantial knowledge about herb pastures.

## Discussion

This paper contributes to theorizing about agricultural innovation by empirically exploring the networks in which farmers discuss science. The investigations reported above focus on three aspects of innovation theory that require development. We need to know more about the organization of local research stakeholders and the roles of facilitation and communication in sustaining the open-ended complexities of agriculture’s innovation systems. Empirically, our investigations have focused on a group of five scientists and 17 farmers. This group has collaboratively undertaken a three-year pastoral farming experiment that produced scientifically new knowledge and was undertaken collaboratively with the farmers as local research stakeholders. Our analysis of this group has focused on its cohesiveness and on how the farmer members have networked the experimental science with the larger field of social contacts they make to enhance their agricultural knowledge. These farmer knowledge exchanges have been analysed both structurally and thematically. The results suggest that farmers deliberate about science in networks that are both substantial and sociologically organized in ways that have significant implications for theorizing about agricultural innovation.

Network analysis shows that over an 18-month period the group’s 17 farmers talked about the pasture experiment with 192 people. Some 40% of these contacts were individuals with whom they already exchanged herb knowledge before the experiment began; the majority had not been identified at the experiment’s launch. This growth in the experiment’s network reach is sociologically organized. Farmers with densely tied and homogenous contacts grew their networks more than did farmers whose contacts are loosely tied and occupationally diverse. Farmer networking was overwhelmingly directed at social peers. Half their pre-existing knowledge contacts were fellow farmers, as were 70% of the new contacts identified at 18 months. These results confirm the power of homophily in social life. The homophily principle holds that contact occurs more frequently between the similar than the dissimilar [33]. The resulting dense networks formed by social peers generate shared understandings that reduce cognitive distance and so enhance the sharing of knowledge [34]. These shared understandings mean that relations between the socially similar are particularly effective when it comes to communicating complex information whose practical value typically relies heavily on tacit knowledge [35].

Herb pasture knowledge is as experimental for farmers as it is for scientists. As stakeholders in the pasture experiment, the 17 farmers shared its numerous uncertainties, ranging from the latest performance results to three years of unusually dry summers and mild autumns. In such open-ended situations, people often share advice and ideas with their social peers. As research on network diffusion has persistently shown, homophily plays an important role in innovation processes. A classic 1950s study, for example, found that innovation is initiated in interpersonal discussions between close colleagues and that such discussions are more likely to be influential when matters are uncertain rather than clear-cut [36]. The network analysis undertaken above similarly suggests that farmer knowledge exchanges are expressions of their social solidarity. This interpretation has been confirmed and extended by an analysis of statements made by the farmers about their most valued contacts for herb pasture knowledge. Thematic analysis shows that networking by the 17 farmers is sustained by a culture that values persons over roles and the experience of particulars over the application of rationalized generalities. Such norms privilege knowledge about and from fellow farmers. Although the experiential character of farmer knowledge has been highlighted by previous research, this is often taken to imply a reliance on intuitions based in individualized and largely subconscious processes [37]. Farmer reasoning, however, is not mysterious; it operates in plain view as the repeated and public sharing of empirical observations.

Analysis of prior personal contacts between the group’s 22 members indicates a cohesive and decentralized network in which everyone is close together. Stakeholder research has suggested that such structures are particularly well suited for long-term planning and collective problem-solving [35]. The history of prior acquaintance between the 17 farmers and five scientists did not generate a sparsely tied entrepreneurial structure; instead it had the typical form of social support networks coordinated by interpersonal trust [38]. The social proximity of the group members is also evident in the extent to which they know people in common. Triadic analysis shows that the farmers and scientists share many alters; this sharing collectively empowers the group to deliver communications about the experiment to single alters from multiple members. Relative to the scientists, the farmers are better placed to deliver such multi-member messages; fellow farmers are also the most likely recipients. These results suggest that the experiment is embedded in strongly organized and decentralized farmer-driven networks that tie the conduct of science to the experience of farming. Following the lead of cognitive anthropology, we dub this expanded field of knowledge agricultural science in the wild [39], [40]. Agricultural science in the wild is an informal field of naturally occurring interpersonal knowledge developed by the self-organizing and open-ended networking of science by farmers. This networking proceeds without the supervision of an overarching authority that conclusively legitimates knowledge and that circulates this knowledge as a controlled activity.

The social character of agricultural science in the wild has significant implications for theorizing about agricultural innovation. As remarked in the introduction, although it is often argued that innovative knowledge requires more than the linear transfer of technology from scientists through extension agents to farmers, no clear alternative models have emerged. Some 20 years of experimenting with Farmer First initiatives have proved inconclusive and in response the search for alternatives has recently turned to the open-ended and heterogeneous interactions that produce complex systems of agricultural innovation. With its characteristic emphasis on actor diversity, this theoretical turn risks underestimating the role played by social homophily in innovation. Our analysis of the 17 farmers’ contact networks reveals structures that are dense, homophilic and decentralized. The farmers’ network culture animates these structures by privileging experience in ways that informally pluralize what counts as authoritative agricultural knowledge. Our analysis thus suggests that the complexity of agricultural innovation is not only the systemic context of farming; it is also a significant outcome of networking by farmers themselves.

The turn to systems theory in studies of agricultural innovation has often been accompanied by calls to professionalize the specialist skills of network facilitation. Given the frequently observed relationship between professionalism and socially closed expertise, we should be cautious about such calls. In any case, our analysis suggests that these new professionals might have little to do because, by and large, they do not play an overarching role in the knowledge flows of complex innovation systems. Our analysis suggests that farmers exchange new scientific knowledge within durable relationships in which they themselves are the principal facilitators. The herb pasture experiment is not at the centre of these relationships. Knowledge did not simply radiate outwards; it was developed in self-organizing and distributed farmer networks. The capacity of distributed networks to produce resilient and action-changing innovations has been well documented in numerous fields [41]. Such social structures self-organize around locally available resources and operate largely independent of materials shipped in from elsewhere. Our analysis thus confirms theoretical observations that communication about new agricultural knowledge is more likely in everyday interactions and conversations than in professionally facilitated meetings and activities [18]. These everyday farming interactions embed actors in networks that are, quantitatively speaking, densely decentralized and, qualitatively speaking, intensely particularised. Rather than extending information from one location to another, knowledge is communicated to the network’s members as they directly engage in its ongoing development. In such networks, participation is critical and the communication of new knowledge is part-and-parcel of its production [42]. On these terms then, the challenge is not to theorize and professionally legitimize new forms of specialist facilitation that link the knowing with the unknowing. Rather, it is to take stock of the farmer-facilitated networking already in practice and to learn more about the complexity it introduces into the systems that sustain agricultural innovation.
